# Supplementary material for: Patient perspectives on physician competence: Validation of the CanMEDS framework
Source: PLOS Glob Public Health. 2025 Dec 30;5(12):e0005716. doi: 10.1371/journal.pgph.0005716 (PMC12753085; doi:10.1371/journal.pgph.0005716)
Supplement: S1 Table — (DOCX) [file pgph.0005716.s001.docx]

**Supplementary file 2: Table 1:** Participant baseline characteristics

| **Characteristic** | **Overall**  N = 403^1^ | **Female**  N = 290^1^ | **Male**  N = 113^1^ | **p-value**^2^ |
| --- | --- | --- | --- | --- |
| **Education Level** |  |  |  | <0.001 |
| Bachelor | 74 (18%) | 55 (19%) | 19 (17%) |  |
| Master | 45 (11%) | 36 (12%) | 9 (8.0%) |  |
| MD | 54 (13%) | 25 (8.6%) | 29 (26%) |  |
| Other | 7 (1.7%) | 5 (1.7%) | 2 (1.8%) |  |
| PhD | 6 (1.5%) | 5 (1.7%) | 1 (0.9%) |  |
| Undergrad | 217 (54%) | 164 (57%) | 53 (47%) |  |
| **Chronic Visit Status** |  |  |  | 0.001 |
| No | 291 (72%) | 196 (68%) | 95 (84%) |  |
| Yes | 112 (28%) | 94 (32%) | 18 (16%) |  |
| **Work Type** |  |  |  | 0.010 |
| Business | 18 (4.5%) | 15 (5.2%) | 3 (2.7%) |  |
| Education | 30 (7.5%) | 24 (8.3%) | 6 (5.4%) |  |
| Engineering | 25 (6.2%) | 14 (4.8%) | 11 (9.8%) |  |
| Healthcare | 218 (54%) | 146 (51%) | 72 (64%) |  |
| Other | 110 (27%) | 90 (31%) | 20 (18%) |  |
| Unknown | 2 | 1 | 1 |  |
| **Region** |  |  |  | 0.8 |
| Mixed | 8 (2.0%) | 5 (1.7%) | 3 (2.7%) |  |
| Rural | 113 (28%) | 83 (29%) | 30 (27%) |  |
| Urban | 282 (70%) | 202 (70%) | 80 (71%) |  |
| **Income Category** |  |  |  | 0.2 |
| < 250 $ | 24 (6.0%) | 18 (6.2%) | 6 (5.3%) |  |
| 250-500 $ | 60 (15%) | 47 (16%) | 13 (12%) |  |
| 500-1000 $ | 161 (40%) | 122 (42%) | 39 (35%) |  |
| 1000-3000 $ | 113 (28%) | 73 (25%) | 40 (35%) |  |
| > 3000 $ | 45 (11%) | 30 (10%) | 15 (13%) |  |
| **Preferred Doctor Age** |  |  |  | 0.5 |
| <40 | 63 (16%) | 42 (14%) | 21 (19%) |  |
| >60 | 4 (1.0%) | 4 (1.4%) | 0 (0%) |  |
| 40-60 | 149 (37%) | 109 (38%) | 40 (35%) |  |
| No Preference | 187 (46%) | 135 (47%) | 52 (46%) |  |
| **Preferred Doctor Gender** |  |  |  | <0.001 |
| Female | 63 (16%) | 60 (21%) | 3 (2.7%) |  |
| Male | 72 (18%) | 42 (14%) | 30 (27%) |  |
| No Preference | 268 (67%) | 188 (65%) | 80 (71%) |  |
| **Preferred Doctor Religion** |  |  |  | 0.7 |
| No | 349 (87%) | 253 (87%) | 96 (85%) |  |
| Yes | 54 (13%) | 37 (13%) | 17 (15%) |  |
| **Preferred Doctor Language** |  |  |  | 0.4 |
| No | 264 (66%) | 186 (64%) | 78 (69%) |  |
| Yes | 139 (34%) | 104 (36%) | 35 (31%) |  |
| **Preferred Hospital Type** |  |  |  | 0.4 |
| No Preference | 128 (32%) | 98 (34%) | 30 (27%) |  |
| Private | 269 (67%) | 188 (65%) | 81 (72%) |  |
| Public | 6 (1.5%) | 4 (1.4%) | 2 (1.8%) |  |
| **Doctor Age** | 49.06 ± 8.35 | 49.52 ± 7.97 | 46.44 ± 10.15 | 0.3 |
| **Medical Expert** | 4.52 ± 0.64 | 4.55 ± 0.59 | 4.44 ± 0.77 | 0.15 |
| **Communicator** | 4.39 ± 0.63 | 4.42 ± 0.60 | 4.31 ± 0.72 | 0.2 |
| **Collaborator** | 4.27 ± 0.78 | 4.31 ± 0.72 | 4.18 ± 0.90 | 0.2 |
| **Leader** | 4.28 ± 0.76 | 4.32 ± 0.71 | 4.18 ± 0.89 | 0.11 |
| **Professional** | 4.33 ± 0.69 | 4.36 ± 0.65 | 4.27 ± 0.78 | 0.3 |
| **Health Advocate** | 4.36 ± 0.75 | 4.38 ± 0.70 | 4.32 ± 0.87 | 0.5 |
| **Scholar** | 4.35 ± 0.71 | 4.39 ± 0.68 | 4.26 ± 0.80 | 0.11 |
| ^1^n (%); Mean ± SD | | | | |
| ^2^Pearson's Chi-squared test; NA; Welch Two Sample t-test | | | | |
