# Supplementary material for: Patient perspectives on physician competence: Validation of the CanMEDS framework
Source: PLOS Glob Public Health. 2025 Dec 30;5(12):e0005716. doi: 10.1371/journal.pgph.0005716 (PMC12753085; doi:10.1371/journal.pgph.0005716)
Supplement: S1 File — (DOCX) [file pgph.0005716.s002.docx]

**Supplementary file 3: Questionnaire**

Your responses will remain anonymous, ensuring your privacy throughout the data collection and analysis process. Your contribution is greatly appreciated.

**Section 1: Informed consent**

1. Please check the below statements to proceed to the survey

- I have read and understood the information provided. I acknowledge that my participation is voluntary, that my data will remain confidential, and I consent to participate in this study
- I don't agree

**Section 2: Sociodemographic characteristics:**

1. Age:
2. Gender

- Male
- Female

1. Highest level of education

- Undergraduate student
- Bachelor’s degree
- Medical degree (MD)
- Master’s degree
- Doctorate (PhD…)
- Other

1. What is your work type?

- Healthcare and Social Services
- Education and Public Services
- Business, Finance, and Administration
- Technical, Engineering, and Skilled Trades
- Retail, Hospitality, and Customer Service
- Other:

1. Living region category

- Rural
- Urban
- Other:

1. Personal monthly income

- No income
- < 250 USD
- 250-500 USD
- 500-1000 USD
- 1000-3000 USD
- > 3000 USD

**Section 3: Current Physician Characteristics**

1. Do you usually consult a family member, friend, or neighbor who is a doctor or a Family Medicine Doctor before deciding to visit a Specialized physician?

- Yes
- No

1. Do you regularly visit a specialist for a chronic condition you have?

- Yes
- No

1. What is the gender of this physician?

- Female
- Male
- I don’t regularly visit a specialized physician

1. How frequently do you see this physician?

- Every 3 months
- Every 6 months
- Every 12 months
- > 1 year
- I don’t regularly visit a specialized physician

1. What is the approximate age of this physician?

If you don’t regularly visit a specialized physician, put zero (0)

**Section 4: Physician Preference**

1. What is your preference for the physician’s age?

- <40
- 40-60
- >60
- No preference

1. What is your preferred gender for the physician?

- Woman
- Man
- No preference

1. Do you have a preference for the religion of the physician?

- Yes
- No
- No preference

1. Do you have a preference for the language spoken by the physician?

- Yes
- No
- No preference

1. Do you prefer receiving medical care at a private hospital or a public (general) hospital?

- Private hospital
- Public hospital
- No preference

1. Where do you typically search for information or seek recommendations about a doctor before scheduling a visit?

- Social media (Facebook, Instagram, etc.)
- Hospital websites or online health directories
- Recommendations from family or friends
- Online reviews on healthcare platforms (e.g., Healthgrades, WebMD)
- Personal experience with the doctor
- Other:

**Section 5: CanMEDS scale**

**Answer the following based on:**

Strongly disagree (1)

Disagree (2)

Neutral (3)

Agree (4)

Strongly Agree (5)

1. **In your opinion a doctor should be a “Medical Expert” such that:**

- Has solid knowledge and applies it to offer the best care
- Is able to get a good medical history, perform a rigorous physical exam, asks for the necessary paraclinical tests, puts the right diagnosis and proposes to the patient a clear management of his illness based on priorities
- Prescribes the appropriate treatment and explains about its side-effects
- Ensures a continuity in the care and treatment of the patient
- Preserves the patient’s safety
- Your doctor explains the evidence or reasoning behind their medical decisions
- Your doctor uses visual aids or diagrams to help explain your condition when needed

1. **In your opinion a doctor should be a “Communicator” such that:**

- Takes time to listen to me
- Does everything to make me feel I can trust him/her
- Explains what the treatment is for
- Takes account of my preferences in prescribing medication
- Gives me the impression he/she has respect for me
- Gives me information on the side effects of medication
- Emphasises which are the most important drugs
- Discusses any difficulties I have in complying with the treatment
- Explains things in simple words
- Offers new treatment
- Writes the prescription legibly
- Lets me ask questions
- Gives me incentives to comply with the treatment
- Gives me advice on prevention (diet, physical activity)
- Gives the impression he/she knows his/her job
- Communicates with the patient and the family with respect and compassion and leads a good Conversation
- Listens to the patient without interrupting and gives the necessary time to get the important information
- Explains to the patient the disease and treatment
- Encourages the patient and the family to ask questions to understand more the disease and take part in the decisions
- Documents all informations while preserving confidentiality

1. **In your opinion a doctor should be a “Health Advocate” such that:**

- Works at the level of patients to ensure the prevention and awareness of diseases
- Works at the level of the community to ensure the prevention and awareness of diseases
- Your doctor discusses how to prevent your condition or maintain your overall health
- Your doctor actively addresses barriers you face in accessing healthcare services (e.g., cost, transportation)

1. **In your opinion a doctor should be a “Collaborator” such that:**

- Collaborates effectively with other healthcare professionals
- Knows how to deal with conflicts and misunderstandings with colleagues
- Is capable of transferring the care of the patient to another colleague if necessary
- Your doctor actively involves other healthcare professionals (e.g., nurses, specialists) to improve your care
- Your doctor involves you and your family in decision-making about your treatment plan

1. **In your opinion a doctor should be a “Professional” such that:**

- Upholds scientific standards and bases decisions on scientific evidence and experience
- Maintains relationships with research subjects that do not exploit personal financial gain, privacy, or sexual advantages
- Takes time to review other colleagues' work and provides meaningful and constructive comments to improve it
- Seeks self improvement
- Reports data consistently, accurately and honestly
- Avoids offensive speech that offers unkind comments and unfair criticisms
- Shows a willingness to initiate and offer assistance toward a colleague's professional and personal development
- Promotes the welfare and development of junior faculty
- Refusal to violate one's personal and professional code of conduct
- Appreciates and respects the diverse nature of research subjects and/or patients, and honors these differences in one's work with them
- Attends faculty meetings, seminars, and student research presentations as a reflection of support
- Works collaboratively and respectfully within a team to the benefit of improved patient care or to the contribution of research
- Participates in corrective action processes toward those who fail to meet professional standards of conduct
- Does not seek to advance one's career at the expense of another's career
- Volunteers one's skills and expertise for the welfare of the community
- Meets commitments and obligations in a conscientious manner
- Respects the rights, individuality, and diversity of thought of colleagues and students
- Meaningfully contributes to the teaching mission of the department and the College of Medicine
- Shows compassion
- Demonstrates adaptability in responding to changing needs and priorities
- Promotes justice in the bio-medical science system by demonstrating efforts to eliminate discrimination in research
- Respects patient autonomy and helps them make informed decisions
- Assumes leadership in research endeavors
- Recognizes one's own limitations
- Assumes personal responsibility for decisions regarding research activities
- Participates in activities aimed at attaining excellence in biomedical science
- Reports medical or research errors
- Acts in ways that show a commitment to confidentiality
- Adopts uniform and equitable standards for research
- Demonstrates empathy
- Advocates the patient's or research subject's interest over one's own interest
- Discloses any conflicts of interest in the course of professional duties and activities
- Is professionally attired in a manner that is respectful of others
- Responds to constructive criticism by working to improve one's capability in the area criticized
- Commits to implement cost-effective research methods
- Represents information and actions in a truthful way
- Acts with his patients with high ethics
- Acts in response to the society’s expectation of professionalism
- Follows the laws of the medical profession
- Preserves his well-being in order to give the best care to patients

1. **In your opinion a doctor should be a “Leader” such that:**

- Applies a policy of improvement in his care for patients
- Ensures best quality with minimal use of resources
- Leads well to ensure best quality of care
- Manages well his time and work
- Your doctor efficiently manages their time during your consultations

1. **In your opinion a doctor should be an “Erudite” such that:**

- Reads regularly and follows a plan for continuous education
- Teaches students without jeopardizing the patient’s safety
- Is up to date in his medical knowledge
- Is involved in research
- Your doctor discusses new treatments or research findings relevant to your condition
- Your doctor participates in clinical trials or research that could benefit patient care

**Section 6: CanMEDS scale categories ranking**

**After reading all the paragraphs from A to G, please put in order the points A, B, C, D, E , F , G, from the most important to the least important qualities of a physician.**

**1 being the most important & 7 being the least important**

A. The Medical Expert is the one who:

B. The Communicator is:

C. The Health Advocate is the one who:

D. The Collaborator is the one who:

E. The Professional is:

F. The Leader is:

G. The Erudite is:
